# Sperm imprinting integrity in seminoma patients?

**DOI:** 10.1186/s13148-018-0559-z

**Published:** 2018-10-19

**Authors:** Céline Bruno, Oxana Blagoskonov, Julie Barberet, Magali Guilleman, Sandrine Daniel, Benjamin Tournier, Christine Binquet, Cécile Choux, Patricia Fauque

**Affiliations:** 1 Université Bourgogne Franche-Comté-Equipe Génétique des Anomalies du Développement (GAD) INSERM UMR1231, 2 Rue Angélique Ducoudray, F-21000 Dijon, France; 2 CHU Dijon Bourgogne, Laboratoire de Biologie de la Reproduction–CECOS, 14 rue Gaffarel, F-21000 Dijon, France; 3 CHRU Besançon, Service de Biologie et de Médecine de la Reproduction–CECOS, 3 Boulevard Fleming, F-25030 Besançon, France; 4 CHU Dijon Bourgogne, Centre d’Investigation Clinique, Module Epidémiologie Clinique/Essais Cliniques (CIC-EC), 7 boulevard Jeanne d’Arc, F-21000 Dijon, France; 5 Université Bourgogne Franche-Comté-INSERM, CIC1432, Module Épidémiologie Clinique, 7 boulevard Jeanne d’Arc, F-21000 Dijon, France; 6 CHU Dijon Bourgogne, Service de Pathologie, 14 rue Gaffarel, F-21000 Dijon, France; 7 CHU Dijon Bourgogne, Service de Gynécologie-Obstétrique, 14 rue Gaffarel, F-21000 Dijon, France

**Keywords:** Testicular germ cell tumor (TGCT), Seminoma, Imprinted genes, Sperm DNA methylation, Testicular dysgenesis syndrome (TDS), Oligozoospermia

## Abstract

**Background:**

Testicular germ cell tumor such as seminoma is strongly associated with male reproductive problems commonly associated with the alteration of sperm parameters as described in testicular dysgenesis syndrome. Interestingly, numerous studies have reported that the precursor of germ cell cancer, germ cell neoplasia in situ (GCNIS), present similarities to fetal gonocytes, specifically characterized by global DNA hypomethylation particularly on imprinting sequences. These disorders may have a common origin derived from perturbations of embryonal programming during fetal development. Presently, there is no available information concerning the sperm DNA methylation patterns of testicular cancer patients. For the first time, we evaluated the sperm imprinting of seminoma patients.

A total of 92 cryopreserved sperm samples were included, 31 before seminoma treatment (S): 23 normozoospermic (SN) and 8 oligozoospermic (SO) and 61 sperm controls samples: 31 normozoospermic (N) and 30 oligozoospermic (O). DNA methylation levels of seven differentially methylated regions (DMRs) of imprinted genes [*H19/IGF2*: IG-DMR (CTCF3 and CTCF6 of *H19* gene); *IGF2*-DMRs (DMR0 and DMR2); *MEG3/DLK1*:IG-DMR; *SNURF:*TSS-DMR; *KCNQ1OT1*:TSS-DMR] were assessed by pyrosequencing. All comparative analyses were adjusted for age.

**Results:**

Comparisons of sperm DNA methylation levels between seminoma (S) and normozoospermic (N) samples showed a significant difference for the *SNURF* sequence (*p* = 0.017), but after taking into account the sperm parameters, no difference was observed. However, we confirmed a significant association between oligozoospermia (O) and imprinting defects for *H19/IGF2-*CTCF6 (*p* = 0.001), *MEG3/DLK1* (*p* = 0.017), *IGF2-*DMR2 (*p* = 0.022), and *SNURF* (*p* = 0.032) in comparison with control groups (N).

**Conclusions:**

This study highlights the high risk of sperm imprinting defects in cases of oligozoospermia and shows for the first time that seminoma patients with normal spermatogenesis present sperm imprinting integrity. These data suggest a low probability of the involvement of a common imprinting defect in fetal cells leading to both TGCT and subfertility.

**Electronic supplementary material:**

The online version of this article (10.1186/s13148-018-0559-z) contains supplementary material, which is available to authorized users.

## Background

It is now well documented that the incidence of testicular germ cell tumor (TGCT) has been increasing over the past decades, particularly in developed countries [1–4]. It could become a real sanitary problem as recent estimations have predicted that in 2025 around one in 100 men will be diagnosed with TGCT [3]. Moreover, the risk of TGCT is strongly associated with several male reproductive problems such as cryptorchidism, hypospadias, disorders of sex development, low testosterone levels, and the alteration of sperm parameters [5–9]. The association of various disorders, defined by Skakkebaeck and colleagues as testicular dysgenesis syndrome (TDS) [8], may have a common origin derived from perturbations of fetal programming [9]. Moreover, the unconventional inheritance for TGCT risk both in humans and mice suggest the involvement of epigenetic mechanisms probably through environmental effects [10].

Numerous studies have reported that the precursor of germ cell cancer, i.e., germ cell neoplasia in situ (GCNIS) [11], presents similarities to fetal gonocytes, such as cellular, epigenetic, and transcriptomic patterns [12–15]. New insights to epigenetics have showed different genome methylation patterns depending on the types of TGCT [16]. Indeed, contrary to non-seminoma, seminoma cells are characterized by low DNA methylation levels, as found for GCNIS [17–21].

Moreover, TGCT patients present more frequently with subfertility and abnormal spermatogenesis [22]. Interestingly, during the last decade numerous studies have reported abnormal sperm DNA methylation in infertile patients especially for oligozoospermic men [23–34]. These methylation defects occur at imprinted loci (mostly for *IGF2* and *H19* genes), and promoters regions as well as genome-wide [25, 35–40].

All of these raise the issue of the reproductive health in TGCT context, especially on the imprinting process which takes place in the germline during fetal development.

For the first time, we have addressed the question of sperm DNA methylation patterns in TGCT patients. We chose to specifically investigate seminoma for its epigenetic pattern similar to GCNIS by performing sperm DNA methylation analyses using pyrosequencing technology on seven imprinted genes. From a total of 92 men included in this study, we showed major sperm imprinting defects in seminoma patients with oligozoospermia as well of those observed for oligozoospermic men.

## Results

Our study included 92 sperm samples from men who had cryopreserved sperm: 31 before seminoma treatments (S), and 61 in the context of ART procedures who served as controls [31 normozoospermic (N) and 30 oligozoospermic (O)]. Among seminoma patients, 23 (74%) were normozoospermic (SN) and 8 (26%) were oligozoospermic (SO). Patient characteristics and sperm parameters in each group are reported in Table 1. Twelve seminoma patients (38%) were known to have had fathered before cryopreservation and 21 (81%) at the time of inclusion, 4 patients had a history of retractile testicles (without cryptorchidism) and 1 presented history of testicular trauma. Concerning N group patients, 81% had at least one child at inclusion and none of them presented uro-genital conditions. For oligozoospermic control men (O group), 68% were known to have fathered at inclusion following ART, 15 (50%) had urogenital issues including cryptorchidism (*n* = 6 patients), retractile testicles (*n* = 2), varicoceles (*n* = 1), testicular torsion or trauma (*n* = 3), mumps orchitis (*n* = 1), inguinal hernia (*n* = 2). No statistical difference was found between SN vs N groups concerning sperm parameters and fertility status (minimum *p* = 0.135; Table 1).

**Table 1:** Characteristics of study participants

	Control normozoospermic	Seminoma Total	Seminoma normozoospermic	Seminoma oligozoospermic	Control oligozoospermic
	N	S	SN	SO	O
Number of men	31	31	23	8	30
Age (y)	37.1 +/− 5.7^a,b,e^	32.2 +/− 6.5^a,d^	32.2 +/− 6.2^b^	32.4 +/− 7.8	32.9 +/− 4.1^d,e^
Urogenital conditions (no., %)	0	5 (16%)	2 (8%)	3 (37%)	15 (50%)
Cryptorchidism	0	0	0	0	6
Others*	0	5	2	3	9
Concentration (million/ml, ±SD)	66.0 +/− 72.3^a,e^	35.9 +/− 33.7^a,d^	46.3 +/− 33.3	6.1 +/− 4.9	4.5 +/− 6.4^d,e^
Count (million, ±SD)	257.4 +/− 335.3^a,e^	129.8 +/− 177.4^a,d^	169.8 +/− 190.7	14.9 +/− 12.3	11.6 +/− 10.2^d,e^
Progressive motility (%, ±SD)	46.3 +/− 13.4^e^	44.4 +/− 20.1^d^	50.3 +/− 15.9	27.5 +/− 22.3	27.9 +/− 15.3^d,e^
Normal morphology (%, ±SD)	31.2 +/− 17.7^e^	32.1 +/− 18.3^d^	31.1 +/− 17.7	36.0 +/− 21.9^c^	6.3 +/− 6.9^c,d,e^
Father at inclusion (%)^f^	81	81	89	50	68

*SD* standard deviation, *y* years

Significant difference between N and S^a^; N and SN^b^; SO and O^c^; O and S^d^; N and O^e^ (*p* < 0.05)

*Retractile testicles, varicocele, testicular torsion, mumps orchitis, and inguinal hernia

^f^Percentage according to available data

**Fig. 1:**
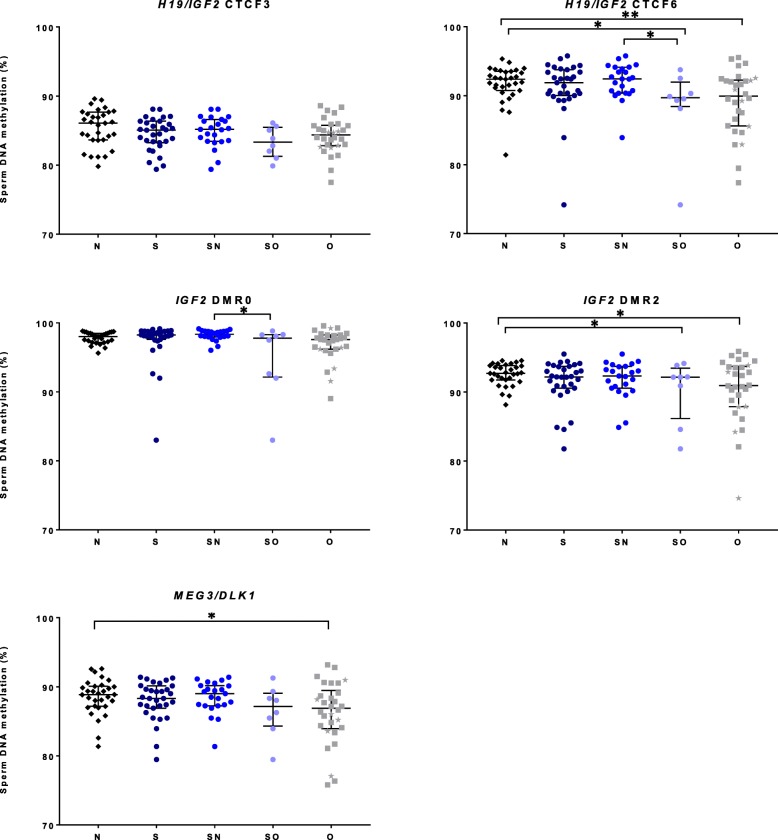
Comparisons of sperm DNA methylation levels between groups on paternal imprinting sequences. Data are represented as median +/− interquartile. Each sample is represented on the graph, as black diamond for normozoospermic (N), dark blue circle for seminoma (S), electric blue circle for seminoma normozoospermic (SN), light blue circle for seminoma oligozoospermic (SO), and gray square for oligozoospermic (O) men. Gray stars refer to data from oligozoospermic men with history of cryptorchidism. *p < 0.05 and **p < 0.01 represent significant differences after adjusting for age

**Fig. 2:**
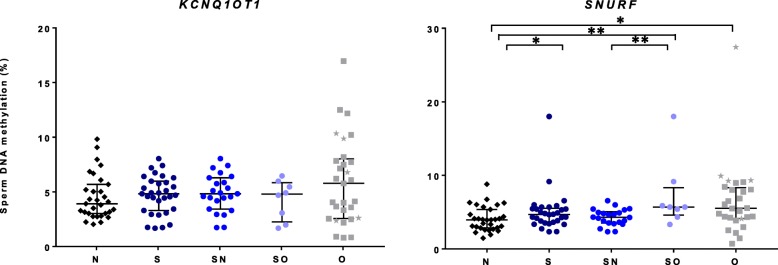
Comparisons of sperm DNA methylation levels between groups on maternal imprinting sequences. Data are represented as median +/− interquartile. Each sample is represented on the graph, as black diamond for normozoospermic (N), dark blue circle for seminoma (S), electric blue circle for seminoma normozoospermic (SN), light blue circle for seminoma oligozoospermic (SO) and gray square for oligozoospermic (O) men. Gray stars refer to data from oligozoospermic men with history of cryptorchidism. *p < 0.05 and **p < 0.01 represent significant differences after adjusting for age

As expected, in the N group, high and low sperm DNA methylation levels were observed for paternal and maternal imprinted genes, respectively (Additional file 1: Table S1A). After adjusting for age, significant differences were found between N vs O groups for *H19/IGF2-*CTCF6 (*p* = 0.001), *MEG3/DLK1* (*p* = 0.017), *IGF2-*DMR2 (*p* = 0.022), and *SNURF* (*p* = 0.032) (Figs. 1 and 2, Additional file 1: Table S1B and Additional file 2: Table S2, Additional file 3: Figure S1). The variability of DNA methylation levels was high in the O group (Fig. 1, Additional file 4: Table S3). Moreover, sperm methylation levels of all O group patients with history of cryptorchidism were identified as outliers for at least one sequence (Fig. 1).

Concerning seminoma patients (S), after adjusting for age, comparisons of sperm DNA methylation levels between the S and N groups showed a significant difference for the *SNURF* sequence (*p* = 0.017). However, after taking into account the sperm parameters, no difference was observed for both SN (seminoma normozoospermic men) vs N and SO (seminoma oligozoospermic men) vs O (Fig. 2, Additional file 1: Table S1B). In contrast, in the SO group, specific imprinting defects were detected for *H19/IGF2-CTCF6* (*p* = 0.013)*, SNURF* (*p* = 0.001) and *IGF2-DMR2* (*p* = 0.019) compared to N group (Figs. 1 and 2, Additional file 1: Table S1B).

## Discussion

To our knowledge, this is the first study to report the analyses of sperm DNA methylation in patients with seminoma. After adjusting for age, imprinting defects on *SNURF* were detected in the seminoma group in comparison with the normozoospermic controls, but after taking into account sperm parameters, we did not observe any significant difference. Therefore, seminoma patients with normal spermatogenesis appear to maintain sperm imprinting integrity. Moreover, our findings confirmed a strong association between oligozoospermia and imprinting defects (herein, on *H19/IGF2*, *MEG3/DLK1*, and *SNURF* DMRs for oligozoospermic patients with or without seminoma).

In this study, we chose to compare normozoospermic and oligozoospermic controls because seminoma patients could have altered sperm parameters. Moreover, 40–50% of TGCT patients present low sperm concentration before cryopreservation [6, 41, 42]. However, in the present study, only 26% of seminoma patients showed signs of oligozoospermia. This difference could be explained by a drastic selection of samples. Herein, we excluded (1) samples with somatic cell contamination at systematic microscopic control of the preparation of purified spermatozoa, (2) samples with altered quality of extracted sperm DNA, (3) patients with extreme oligozoospermia.

Our analyses on controls allowed us to confirm imprinted defects in samples with low sperm concentration [33]. As previously reported, we observed altered sperm DNA methylation for oligozoospermic controls in the *H19-IGF2*, *IGF2-*DMR2, *SNURF*, and *MEG3/DLK1* DMRs [23, 26]*.*

All these alterations both on maternal and paternal imprinted genes (hyper- and hypomethylation, respectively) observed in “control” cases of deficient spermatogenesis, could be due to failures in erasure/gain of DNA methylation during germ cell development [23]. Moreover, the association of cryptorchidism with sperm imprinting defects observed in the present study may be in favor of the hypothesis of a fetal origin to these defects.

Since the 1990s, most epidemiological trends have reported a decrease of semen parameters in developed countries [43], associated with an increased risk of male reproductive disorders like cryptorchidism, hypospadias, disorders of sex development, and TGCT [9, 44]. Moreover, given these observations and based on epigenetic findings, environmental influences during fetal life are largely suspected in the occurrence of testicular dysgenesis syndrome (TDS). Indeed, it has been reported in animal models that the exposition to endocrine disruptors during fetal life lead to testicular diseases, sperm parameter alterations, obesity, and sperm epigenetic defects on imprinted genes [45–51]. However, at present, there is no available information about the epigenetic status of the human germ cells in the TDS environment and particularly in TGCT. To address this issue, we performed analyses on sperm both in subfertile patients and in seminoma patients. Notably, seminoma cells presented epigenetic patterns close to GCNIS and were characterized by a global DNA hypomethylation, particularly on imprinting sequences [18, 20, 21].

We found that seminoma patients with oligozoospermia also presented sperm DNA methylation alterations on imprinted genes. The alterations were quite similar to those observed in oligozoospermic controls.

These observations suggest (1) that spermatozoa in the seminoma environment are not systematically associated with imprinting defects and, (2) a low probability of the involvement of a common imprinting defect in fetal germ cells leading to both TCGT and subfertility.

One hypothesis is that two independent fetal processes could be involved; one in germ cells dedifferentiation leading to GCNIS formation and the second leading to imprinting defects in germ cells. The occurrence of one or the other or both may depend on the time, intensity, and the period of exposition to an inappropriate fetal environment. Another explanation of the increase in sperm parameters alterations in the TGCT context could be the consequences of tumor proliferation and local inflammation.

Indeed, presently emerging evidence highlight the influence of environmental factors on human sperm DNA methylation, notably lifestyle factors [52–56]. As hypothesized by Kobayashi et al., the relation between paternal age and sperm DNA methylation abnormalities could be the memory of cumulative environmental influences over the years [55]. Interestingly, Jenkins and al. used microarray analyses to identify regions which were significantly hypomethylated (*n* = 139) and hypermethylated (*n* = 8) in advanced-age paternal sperm [53]. However, none of the imprinted genes analyzed in our study was identified to be age-susceptible.

Altogether, these results are rather reassuring in regards to the sperm imprinting integrity of seminoma patients as well as their fertility. Indeed, in the present study, 84% of seminoma patients were known to have fathered at the time of inclusion, in accordance with the few clinical reports [22, 57]. However, there may be epigenetic risks linked to sperm alterations [6, 41, 42]. As most seminoma patients are young and have not fathered at the time of sperm cryopreservation (herein 62%), the question on the association with potential imprinting defects and their consequences for the conceptus is particularly crucial. Indeed, it is already known that assisted reproductive technologies (IVF or ICSI), widely used for men with oligozoospermia, are associated with increased incidences of rare imprinting disorders, especially Beckwith-Widemann syndrome, Angelman syndrome and Silver-Russell syndrome. The technique itself could be involved [58], but the use of sperm with preexisting imprinting defects is not excluded. Moreover, a correlation between sperm methylation alterations and adverse pregnancy outcomes have also been reported [26, 55, 59, 60]. However, these germ cell DNA methylation defects could have consequences later in life, leading to various diseases in adulthood such as cardiovascular diseases or cancers [61, 62].

There are some limitations in this study. First, although somatic cells were not observed during microscopic control of all sperm preparations, an improbable somatic cell contamination cannot be fully excluded. Nevertheless, we reported comparable sperm DNA methylation levels for oligozoospermic men as observed in previous studies [23, 56]. Second, the pyrosequencing method allowed us to analyze DNA methylation at only a few CpGs per DMR (between 3 and 18). Even though it would have been interesting to perform analyses at larger scale, the resolution in imprinted regions would have been lower. And third, as previously discussed, a lack of information concerning the potential confounding factors could modify the results, though the adjustment for age limited this effect.

Overall, it still remains to be determined whether seminoma patients with alterations to spermatogenesis can return to spermatogenesis and DNA methylation levels comparable to the normozoospermic and fertile control population on the tested sequences and also on large scale.

## Conclusions

This study highlights the high risk of sperm imprinting defects (both in paternal and maternal DMRs) in cases of oligozoospermia and shows that seminoma patients with normal spermatogenesis presented sperm imprinting integrity. These novel data disprove the idea of a common fetal imprinting defect in germ cells leading to both CGNIS and subfertility. Environmental factors are strongly suspected and could act throughout life, even if the fetal period is presumptively more at risk for the development of severe chronic diseases.

Our findings shed light onto the need to set up epigenetic explorations for men with spermatogenesis deficiencies.

## Methods

### Aim, design, and setting of the study

To assess the sperm imprinted pattern of seminoma patients, a total of 92 sperm samples were included 31 before seminoma treatment (S) and 61 in the context of ART procedures who served as controls: 31 normozoospermic (N) and 30 oligozoospermic (O). Among seminoma patients (S) (*n* = 31), we established two subgroups according to their sperm parameters: normozoospermic seminoma patients (SN) (*n* = 23) and oligozoospermic seminoma patients (SO) (*n* = 8). Seminoma samples were selected according to the histology analyses, and only cryopreserved sperm from pure seminoma patients were included in the study.

We specifically selected imprinted genes which are known to be perturbed in altered spermatogenesis [23, 33] and/or in a context of infertility [24, 27, 33]. DNA methylation levels of seven differentially methylated regions (DMRs) of imprinted genes (*H19/IGF2*:IG-DMR: CTCF3 and CTCF6 of *H19* gene and the DMR0 and DMR2 of *IGF2* gene; *GTL2*-DMR; *SNURF:*TSS-DMR; *KCNQ1OT1*:TSS-DMR) were assessed by pyrosequencing. All analyses were performed after adjusting for age and compared according to the sperm parameters.

### Subjects and samples

Cryopreserved sperm samples from 92 patients were collected from two CECOS centers (Centre d’Etude et de Conservation des Oeufs et du Sperm) in Dijon and Besançon, France. According to French law, patients who have cryopreserved sperm and want to dispose of it can choose to donate their sperm straws to research programs. All volunteer patients provided written informed consent as approved by the Ethics Committee of the University and the state medical board (2016 PHRCI-16-046, NCT03262207). Thirty-one samples were obtained from patients who cryopreserved their sperm before seminoma treatments (S), 30 from oligozoospermic (O) patients who underwent undergone fertility treatments, and 31 from normozoospermic patients (N) who cryopreserved before vasectomy or egg donation attempts. In all groups, samples were selected according to sperm parameters with at least 0.5 M/ml for sperm concentration, and no leucocytes or others cells identified before cryopreservation in order to limit diploid cell contamination.

### Sperm cryopreservation protocol

After 30 min of liquefaction, fresh ejaculate was diluted 1:1 in sperm cryoprotector medium (Spermfreeze™, Vitrolife) and filled in straws. Samples were frozen using programming controlled rate freezer (Minicool LC40 Air liquid or Kryo 560-Planer PLC) and cooled from 4 °C to − 8 °C at a rate of − 5 °C/min, then at a rate of − 10 °C/min to − 25 °C and finally at rate of − 25 °C/min to − 140 °C. Samples were then plunged into nitrogen for storage.

### Thawing procedure and sperm preparation

Thawing was performed in an incubator system at 37 °C during 7 min. Spermatozoa were purified using a Percoll gradient with two concentration layers (90/45%, PureSperm, JCD) to remove lymphocytes, epithelial cells, cell debris, bacteria, abnormal spermatogenic cells, and seminal fluid. Sperm purity was controlled by inverted light microscopy and samples were then stored at − 80 °C until further use.

Measurements of sperm count, sperm motility, and sperm morphology were assessed in accordance with the World Health organization (WHO) guidelines both before and after freezing.

### DNA extraction

Sperm DNA was extracted according to the modified protocol described by Marques et al. [28]. Briefly, sperm pellets were overlaid with 500 μl of lysis solution (LS) containing 10 mM Tris-HCl pH 7.5, 10 mM EDTA, 0.2% SDS, 50 mM NaCl, 1 mM DTT, and 0.2 mg/ml proteinase K (Fermentas) and incubated overnight at 55 °C. Sperm DNA was isolated by standard phenol chloroform extraction. DNA was precipitated using cold ethanol (Sigma-Aldrich), washed, and solubilized in water. All DNA samples were quantified/qualified using a Nanodrop Spectrophotometer (Invitrogen). Sperm DNA samples were included in the study if the ratio DO260/280 was between 1.8 and 2 and the DNA concentration above 50 ng/μl.

### DNA methylation analyses

DNA methylation levels of seven differentially methylated regions (DMRs) of imprinted genes [*H19/IGF2*: IG-DMR (CTCF3 and CTCF6 of *H19* gene); *IGF2*-DMRs (DMR0 and DMR2); *MEG3/DLK1*: IG-DMR; *SNURF:*TSS-DMR; *KCNQ1OT1*:TSS-DMR] were assessed by pyrosequencing after sodium bisulfite DNA treatment. Genomic DNA (500 ng) was modified by sodium bisulfite treatment using the EpiTect kit (Qiagen). Bisulfite-treated DNA (25 ng) was subsequently used as the template for PCR amplification prior to pyrosequencing as previously described in Bruno et al., 2015 [63]. Primers are available in Additional file 5: Table S4. Pyrosequencing reactions were performed in the PyroMark Q24 System (Qiagen) with the PyroGold SQA reagent kit according to the manufacturer’s instructions (Pyrosequencing AB, Uppsala, Sweden). The biotinylated PCR products were purified and denatured using the Pyrosequencing Vacuum Prep Tool (Qiagen). Pyrosequencing was performed on a Pyrosequencer Q24 (Qiagen). The DNA methylation level was calculated as the ratio of the C to T peaks at a given CpG site in pyrograms using Pyromark Q24 Software v.2.0.6 (Qiagen). Considering the presence of SNPs and high variability on one CpG of *H19/IGF2-*CTCF6 (no. 5) and two CpGs of *IGF2*-DRMR2 (no. 8 and 9), these CpGs were not considered for quantitative methylation analysis.

### Statistical analyses

Continuous variables are described as median and interquartile range (IQR) or mean ± standard error of the mean (SEM) according to their distribution. Categorical variables are described using percentages. Baseline demographic and clinical characteristics were compared among the five groups depending on cryopreservation indication and sperm parameters. The distribution of continuous variables were compared using Mann-Whitney or Kruskal-Wallis tests and the ones of categorical variables using chi-square test or Fisher exact test when appropriate. Values that did not lie within the interquartile range and above 75th percentile for paternal imprinted genes or below the 25th percentile for the maternal imprinted genes were defined as outliers. Multivariate linear regression analyses were used to adjust all estimated for age. A log transformation of DNA methylation levels was applied to normalize their distribution. All statistical analyses were performed with SAS software, v9.4 (SAS Institute Inc., USA). A two-tailed *p* < 0.05 was considered significant.

## Additional files

Additional file1: **Table S1.** Sperm DNA methylation analyses on imprinted genes for each analyzed group. (DOCX 28 kb)

Additional file 2: **Table S2.** Comparison of sperm DNA methylation between oligozoospermic controls (O) and normozoospermic controls (N), after adjusting for age, for each CpG site of the deregulated imprinted genes. (DOCX 29 kb)

Additional file 3: **Figure S1.** Comparisons of sperm DNA methylation levels between normozoospermic and oligozoospermic controls at each CpG of the specific altered imprinted sequences detected in the study. Methylation levels at each CpG position are expressed in percentage as mean ± SEM. *p < 0.05 and **p < 0.01 significant differences after adjusting for age. N: normozoospermic (black square), O oligozoospermic (gray circle). (DOCX 346 kb)

Additional file 4: **Table S3.** Relative standard deviation (RSD) of sperm DNA methylation on imprinted genes for control groups (normozoospermic and oligozoospermic). (DOCX 15 kb)

Additional file 5: **Table S4.** Primers for pyrosequencing [23, 63, 64]. (DOCX 28 kb)

## Abbreviations

ART: Assisted reproductive technology
DMR: Differentially methylated regions
GCNIS: Germ cell neoplasia in situ
ICSI: Intracytoplasmic sperm injection
IVF: In vitro fertilization
N: Normozoospermic control
O: Oligozoospermic control
S: Seminoma patient
SN: Normozoospermic seminoma patient
SO: Oligozoospermic seminoma patient
TDS: Testicular dysgenesis syndrome
TGCT: Testicular germ cell tumor
