# Supplementary material for: Sperm imprinting integrity in seminoma patients?
Source: Clin Epigenetics. 2018 Oct 19;10:125. doi: 10.1186/s13148-018-0559-z (PMC6194738; doi:10.1186/s13148-018-0559-z)
Supplement: Supplementary file 1 — Table S1. Sperm DNA methylation analyses on imprinted genes for each analyzed group. (DOCX 28 kb) [file 13148_2018_559_MOESM1_ESM.docx]

**Additional file 1:** **Table S1. Sperm DNA methylation analyses on imprinted genes for each analyzed group**

A. Median and interquartile for each group

| *Imprinted genes* | Paternal (P)  Maternal (M) | **N** | **S** | **SN** | **SO** | **O** |
| --- | --- | --- | --- | --- | --- | --- |
| ***H19/IGF2*-CTCF3** | P | 86.1 [83.6-87.7] | 85.1 [83.2-86.4] | 85.2 [83.4-86.6] | 83.3 [81.5-85.3] | 84.4 [82.8-85.7] |
| ***H19/IGF2*-CTCF6** | P | 92.4 [90.7-93.5] | 91.9 [90.0-93.7] | 92.4 [90.4-94.1] | 89.7 [88.7-91.4] | 90.6 [85.6-92.2] |
| ***IGF2* DMR0** | P | 98.0 [97.2-98.5] | 98.2 [97.6-98.8] | 98.4 [97.9-98.8] | 97.8 [92.3-98.3] | 97.6 [96.2-98.2] |
| ***IGF2* DMR2** | P | 92.7 [91.2-93.8] | 91.8 [89.8-93.7] | 92.3 [89.8-93.7] | 91.5 [87.0-93.0] | 90.5 [86.9-93.4] |
| ***MEG3/DLK1*** | P | 88.8 [87.2-90.1] | 88.3 [86.9-90.1] | 89.0 [87.2-90.2] | 87.1 [84.7-88.8] | 86.9 [84.1-89.1] |
| ***KCNQ1OT1*** | M | 3.9 [3.0-5.7] | 4.8 [3.3-5.9] | 4.8 [3.4-6.30] | 4.8 [2.5-5.7] | 5.8 [2.6-7.8] |
| ***SNURF*** | M | 3.9 [2.8-5.4] | 4.7 [3.6-5.5] | 4.3 [3.5-4.9] | 5.7 [4.9-7.5] | 5.5 [4.2-8.2] |

B. Statistical results according to different group comparisons after adjusting for age

|  | **S vs N** | | | | |  | **N vs O** | | | | |
| --- | --- | --- | --- | --- | --- | --- | --- | --- | --- | --- | --- |
| *Imprinted genes* | β | SE | [95% CI] | | *p* |  | β | SE | [95% C I] | | *p* |
| ***H19/IGF2*-CTCF3** | -0.003 | 0.004 | -0.010 | 0.004 | *0.360* |  | 0.006 | 0.004 | -0.001 | 0.014 | *0.100* |
| ***H19/IGF2*-CTCF6** | -0.004 | 0.005 | -0.135 | -0.014 | *0.385* |  | -0.017 | 0.005 | -0.274 | -0.008 | ***0.001*** |
| ***IGF2* DMR0** | 0.000 | 0.008 | -0.016 | 0.016 | *0.995* |  | 0.001 | 0.008 | -0.015 | 0.017 | *0.874* |
| ***IGF2* DMR2** | -0.065 | 0.004 | -0.014 | 0.001 | *0.086* |  | -0.010 | 0.004 | -0.191 | -0.016 | ***0.022*** |
| ***MEG3/DLK1*** | -0.002 | 0.004 | -0.010 | 0.005 | *0.575* |  | 0.013 | 0.005 | 0.002 | 0.023 | ***0.017*** |
| ***KCNQ1OT1*** | 0.000 | 0.051 | -0.102 | 0.102 | *0.997* |  | -0.078 | 0.078 | -0.235 | 0.079 | *0.323* |
| ***SNURF*** | 0.118 | 0.048 | 0.022 | 0.213 | ***0.017*** |  | -0.154 | 0.070 | -0.294 | -0.014 | ***0.032*** |
|  | **SN vs N** | | | | |  | **SO vs O** | | | | |
| ***H19/IGF2*-CTCF3** | -0.001 | 0.004 | -0.008 | 0.007 | *0.875* |  | -0.005 | 0.005 | -0.015 | 0.005 | *0.338* |
| ***H19/IGF2*-CTCF6** | -0.001 | 0.004 | -0.008 | 0.007 | *0.874* |  | -0.004 | 0.010 | -0.024 | 0.016 | *0.698* |
| ***IGF2* DMR0** | 0.005 | 0.009 | -0.012 | 0.023 | *0.556* |  | -0.010 | 0.006 | -0.022 | 0.002 | *0.086* |
| ***IGF2* DMR2** | -0.003 | 0.003 | -0.010 | 0.004 | *0.352* |  | -0.002 | 0.009 | -0.020 | 0.015 | *0.785* |
| ***MEG3/DLK1*** | 0.001 | 0.004 | -0.006 | 0.009 | *0.702* |  | 0.001 | 0.009 | -0.017 | 0.020 | *0.873* |
| ***KCNQ1OT1*** | 0.026 | 0.055 | -0.084 | 0.135 | *0.639* |  | -0.076 | 0.133 | -0.346 | 0.194 | *0.573* |
| ***SNURF*** | 0.057 | 0.048 | -0.039 | 0.153 | *0.241* |  | 0.079 | 0.116 | -0.157 | 0.316 | *0.501* |
|  | **SO vs N** | | | | |  | **SN vs SO** | | | | |
| ***H19/IGF2*-CTCF3** | -0.010 | 0.006 | -0.217 | 0.001 | *0.081* |  | -0.008 | 0.005 | 0.180 | 0.002 | *0.098* |
| ***H19/IGF2*-CTCF6** | -0.195 | 0.007 | -0.346 | -0.004 | ***0.013*** |  | -0.018 | 0.008 | -0.034 | -0.003 | ***0.024*** |
| ***IGF2* DMR0** | -0.014 | 0.015 | -0.443 | 0.017 | *0.364* |  | -0.016 | 0.052 | -0.264 | -0.052 | ***0.005*** |
| ***IGF2* DMR2** | -0.013 | 0.005 | -0.025 | -0.002 | ***0.019*** |  | -0.085 | 0.007 | -0.023 | 0.006 | *0.243* |
| ***MEG3/DLK1*** | -0.011 | 0.006 | -0.230 | 0.001 | *0.071* |  | -0.010 | 0.006 | -0.216 | 0.002 | *0.087* |
| ***KCNQ1OT1*** | -0.025 | 0.081 | -0.190 | 0.139 | *0.757* |  | -0.666 | 0.071 | -0.212 | 0.079 | *0.357* |
| ***SNURF*** | 0.262 | 0.754 | 0.109 | 0.415 | ***0.001*** |  | 0.178 | 0.063 | 0.049 | 0.307 | ***0.009*** |

β coefficients correspond to the mean additional log10 DNA methylation level in patients with the given characteristics (e.g. oligozoospermic) vs the control group

N: Normozoospermic control group; S: Seminoma group ; SN: Normozoospermic Seminoma patients ; SO: Oligozoospermic Seminoma patients; O: Oligozoospermic control group

*p*<0.05 in bold
