# Supplementary material for: Sperm imprinting integrity in seminoma patients?
Source: Clin Epigenetics. 2018 Oct 19;10:125. doi: 10.1186/s13148-018-0559-z (PMC6194738; doi:10.1186/s13148-018-0559-z)
Supplement: Supplementary file 2 — Table S2. Comparison of sperm DNA methylation between oligozoospermic controls (O) and normozoospermic controls (N), after adjusting for age, for each CpG site of the deregulated imprinted genes. (DOCX 29 kb) [file 13148_2018_559_MOESM2_ESM.docx]

**Additional file 2: Table S2. Comparison of sperm DNA methylation between** **oligozoospermic controls (O) and normozoospermic controls (N), after adjusting for age, for each CpG site of the deregulated imprinted genes**

|  |  | **β** | **[95% CI]** | | ***p*** |  |  | **β** | **[95% CI]** | | ***p*** |
| --- | --- | --- | --- | --- | --- | --- | --- | --- | --- | --- | --- |
| ***IGF2*-DMR2** | **CG1** | -0.017 | -0.039 | 0.004 | *0.108* | ***H19/IGF2*-CTCF6** | **CG1** | -0.156 | -0.029 | -0.015 | ***0.030*** |
|  | **CG2** | -0.020 | -0.040 | -0.001 | ***0.049*** |  | **CG2** | -0.030 | -0.057 | -0.001 | ***0.040*** |
|  | **CG3** | -0.012 | -0.058 | 0.035 | *0.613* |  | **CG3** | -0.014 | -0.024 | -0.005 | ***<0.01*** |
|  | **CG4** | -0.018 | -0.037 | 0.001 | *0.061* |  | **CG4** | -0.021 | -0.037 | -0.006 | ***<0.01*** |
|  | **CG5** | -0.021 | -0.068 | 0.026 | *0.368* |  | **CG5** | -0.010 | -0.026 | 0.001 | *0.080* |
|  | **CG6** | -0.005 | -0.027 | 0.017 | *0.635* |  |  |  |  |  |  |
|  | **CG7** | -0.020 | -0.039 | -0.001 | ***0.045*** | ***MEG3/DLK1*** | **CG1** | -0.002 | -0.017 | 0.013 | *0.778* |
|  | **CG8** | -0.004 | -0.014 | 0.007 | *0.428* |  | **CG2** | -0.006 | -0.017 | 0.006 | *0.352* |
|  | **CG9** | -0.002 | -0.012 | 0.007 | *0.632* |  | **CG3** | -0.008 | -0.017 | 0.001 | *0.075* |
|  | **CG10** | -0.006 | -0.026 | 0.138 | *0.575* |  | **CG4** | -0.015 | -0.035 | 0.006 | *0.153* |
|  | **CG11** | -0.008 | -0.025 | 0.008 | *0.317* |  | **CG5** | -0.021 | -0.051 | 0.010 | *0.183* |
|  | **CG12** | -0.005 | -0.019 | 0.008 | *0.482* |  |  |  |  |  |  |
|  | **CG13** | -0.006 | -0.018 | 0.007 | *0.366* | ***SNURF*** | **CG1** | 0.222 | 0.031 | 0.414 | ***0.024*** |
|  | **CG14** | -0.005 | -0.018 | 0.009 | *0.501* |  | **CG2** | 0.065 | -0.111 | 0.240 | *0.464* |
|  | **CG15** | -0.028 | -0.050 | -0.007 | ***0.011*** |  | **CG3** | -0.010 | -0.198 | 0.177 | *0.913* |
|  | **CG16** | -0.010 | -0.031 | 0.012 | *0.383* |  | **CG4** | 0.145 | -0.044 | 0.335 | *0.130* |
|  | **CG17** | -0.002 | -0.010 | 0.006 | *0.676* |  | **CG5** | 0.036 | -0.168 | 0.239 | *0.726* |
|  | **CG18** | -0.001 | -0.003 | 0.002 | *0.490* |  | **CG6** | 0.153 | -0.202 | 0.327 | *0.052* |
|  |  |  |  |  |  |  | **CG7** | 0.201 | -0.005 | 0.407 | *0.054* |

β Coefficients correspond to the mean additional log10 DNA methylation level in oligozoospermic controls vs normozoospermic controls
