# Supplementary material for: Sperm imprinting integrity in seminoma patients?
Source: Clin Epigenetics. 2018 Oct 19;10:125. doi: 10.1186/s13148-018-0559-z (PMC6194738; doi:10.1186/s13148-018-0559-z)
Supplement: Supplementary file 3 — Figure S1. Comparisons of sperm DNA methylation levels between normozoospermic and oligozoospermic controls at each CpG of the specific altered imprinted sequences detected in the study. Methylation levels at each CpG position are expressed in percentage as mean ± SEM. *p < 0.05 and **p < 0.01 significant differences after adjusting for age. N: normozoospermic (black square), O oligozoospermic (gray circle). (DOCX 346 kb) [file 13148_2018_559_MOESM3_ESM.docx]

Additional file 3

A

*****

*****

*****

*****

*****

******

******

*****
