# Supplementary material for: Sperm imprinting integrity in seminoma patients?
Source: Clin Epigenetics. 2018 Oct 19;10:125. doi: 10.1186/s13148-018-0559-z (PMC6194738; doi:10.1186/s13148-018-0559-z)
Supplement: Supplementary file 4 — Table S3. Relative standard deviation (RSD) of sperm DNA methylation on imprinted genes for control groups (normozoospermic and oligozoospermic). (DOCX 15 kb) [file 13148_2018_559_MOESM4_ESM.docx]

**Additional file 4: Table S3. Relative standard deviation (RSD) of sperm DNA methylation on imprinted genes for control groups (Normozoospermic and Oligozoospermic)**

| ***Imprinted genes*** | **N group** | | |  | **O group** | | |
| --- | --- | --- | --- | --- | --- | --- | --- |
|  | **Mean** | **SD** | **RSD (%)** |  | **Mean** | **SD** | **RSD (%)** |
| ***H19/IGF2*-CTCF3** | 85.4 | 2.7 | 3.2 |  | 84.2 | 2.5 | 3.1 |
| ***H19/IGF2*-CTCF6** | 91.7 | 2.7 | 2.9 |  | 89.2 | 4.6 | 5.2 |
| ***IGF2* DMR0** | 96.6 | 6.9 | 0.8 |  | 96.9 | 2.4 | 2.5 |
| ***IGF2* DMR2** | 92.2 | 1.7 | 1.9 |  | 90.1 | 4.2 | 4.6 |
| ***MEG3/DLK1*** | 88.6 | 2.6 | 2.9 |  | 86.2 | 4.5 | 5.2 |
| ***KCNQ1OT1*** | 4.5 | 2.1 | 45.5 |  | 6.0 | 4.0 | 66.6 |
| ***SNURF*** | 4.1 | 1.7 | 40.8 |  | 6.5 | 4.8 | 74.6 |

N: Normozoospermic control group, O: Oligozoospermic control group, SD: Standard Deviation
