# Supplementary material for: Sperm imprinting integrity in seminoma patients?
Source: Clin Epigenetics. 2018 Oct 19;10:125. doi: 10.1186/s13148-018-0559-z (PMC6194738; doi:10.1186/s13148-018-0559-z)
Supplement: Supplementary file 5 — Table S4. Primers for pyrosequencing [23, 63, 64]. (DOCX 28 kb) [file 13148_2018_559_MOESM5_ESM.docx]

**Additional file 5: Table S4. Primers for pyrosequencing**

| **DMRs** | **Genomic coordinates (hg38)** | **PCR primers** | **Length**  **(bp)** | **HT** | **Sequencing primers** | **CpG sites**  **analyzed** | **References** |
| --- | --- | --- | --- | --- | --- | --- | --- |
| ***H19/IGF2*- CTCF3** | hg38: chr11:1,997,582-2,003,510 | F: TTGGTAGGTATAGAAATTGGGG | 214 | 58°C | seq1 : GTAGTATATGGGTATTTGTG | 3 (2-4) | Boissonnas et al, 2010 (23) |
|  |  | R: biot-ACACCTAACTTAAATAACCCAAAA |  |  | seq2 : GTGGATTTAAAAGTGGT | 7 (5-12) |  |
| ***H19/IGF2-* CTCF6** | hg38: chr11:1,999,843-2,000,081 | F: TGGGTATTTTTGGAGGTTTTTTT | 216 | 52°C | AACTATAATATATAAACCTACAC | 5 (1,3-6) | Boissonnas et al, 2010 (23) |
|  |  | R: CCCATAAATATCCTATTCCCAAAT |  |  |  |  |  |
| ***IGF2-* DMR0** | hg38 : chr11:2,148,240-2,148,320 | F: AGGGGGTTTATTTTTTTAGGAAGT | 81 | 56°C | GGGTTTATTTTTTTAGGAAGTAT | 3 (1-3) | Boissonnas et al, 2010 (23) |
|  |  | R: biot-AAAACCACTAAACACACAACTCTACTTAA |  |  |  |  |  |
| ***IGF2* -DMR2** | hg38 : chr11:2,133,493-2,133,747 | F: GGGAAAGGGGTTTAGGATTTTTAT | 255 | 60°C | seq1: GGTTTAGGATTTTTAT | 7 (1-7) | Boissonnas et al, 2010 (23) |
|  |  | R : biot-ATAATTTACTCCCCCTTCAACCTC |  |  | seq2: GGGTAGATAAGTTTTTTT | 2 (10-11) |  |
|  |  |  |  |  | seq3: TGAGGAAATAGTATTTTTTA | 6 (12-17) |  |
|  |  |  |  |  | seq4: GGAAGTTTTATAGTATAGAG | 3 (18-20) |  |
| ***MEG3/DLK1*** | hg38 : chr14:100,809,344-100,809,438 | F: biot-ATTGAATTGGGTTTGTTAGTAGT | 95 | 56°C | CCTTTATAACAAATTAAAATATATC | 5 (1-5) | Bruno et al, 2015 (41) |
|  |  | R: ATCAAAACAACTCAAATCCTTTATAAC |  |  |  |  |  |
| ***KCNQ1OT1*** | hg38 : chr11:2,700,680-2,700,920 | F: GGTGGTAGGGATATGTTAGGTTATTTA | 121 | 56°C | GGTTATTTATTTGGTAAAGGGTA | 9 (1-9) | Bruno et al, 2015 (41) |
|  |  | R: AAAAACTTTTATAACCCAAACTTTTATCC |  |  |  |  |  |
| ***SNURF*** | hg38 : chr15:24,954,857-24,956,829 | F: GGGAGGGAGTTGGGATTTTTG | 220 | 56°C | AGTTGGGATTTTTGTATTG | 7 (1-7) | Rancourt et al, 2012 (64) |
|  |  | R: biot-AAACCACCCACACAACTAACCTTACCC |  |  |  |  |  |

PCR reaction and pyrosequencing conditions : Pyromark PCR Master Mix 2x 12.5 µL, CoralLoad concentrate 2.5 µL, Primer A 0.2 µM, Primer B 0.2 µM, Water 8 µL, Bisulphite-treated DNA 1 µL. PCR purification: 15 µL PCR product, 40 µL Binding Buffer, 29 µL Water, 1 µL Streptavidin Sepharose High Performance Beads, GE Healthcare, Life Sciences®.

HT : hybridation temperature
